# Supplementary material for: A Likelihood Approach for Real-Time Calibration of Stochastic Compartmental Epidemic Models
Source: PLoS Comput Biol. 2017 Jan 17;13(1):e1005257. doi: 10.1371/journal.pcbi.1005257 (PMC5240920; doi:10.1371/journal.pcbi.1005257)
Supplement: S1 File — (TAR.GZ) [file pcbi.1005257.s014.tar.gz › HSPH_Online-SI-Revision/PF08/n10-extreme/n10-extreme_PF_table8.pdf]

1	R0	{2.68692, 12.5884}	{2.91246, 19.3884}	{{2.9203, 20.}, {3., 20.}}	{12.0433, 55.3362}	inf	138.	571.706	{534.06, 626.61}	314.28	0.606503	newly	61.	176.49	{153., 199.}	189.328	0.456865	Reff	0.251496	0.229662	{0.201967, 0.232625}	18.8281	ar	9089.	11 071.2	{11 025., 11 219.}	21.8082
2	R0	{1.79802, 12.5412}	{2.11829, 19.6719}	{{1.87963, 17.7661}, {2.21648, 20.}}	{17.9396, 56.8585}	inf	348.	728.54	{630.934, 803.064}	109.351	0.318132	newly	172.	235.49	{208., 264.}	36.9128	0.134379	Reff	0.525561	0.480918	{0.446406, 0.536913}	10.1369	ar	7412.	9548.19	{9045., 9777.}	28.8207
3	R0	{2.6174, 10.1105}	{2.98896, 17.9826}	{{3., 17.8463}, {3., 18.2353}}	{14.1956, 77.8606}	inf	48.	377.717	{364.296, 392.235}	686.909	0.895513	newly	29.	126.21	{113., 137.}	335.207	0.637439	Reff	0.238183	0.175932	{0.17111, 0.177638}	26.1359	ar	9109.	10 738.5	{10 680., 10 786.}	17.8884
4	R0	{2.27532, 14.3149}	{2.12108, 18.7526}	{{2.05391, 17.1102}, {2.22245, 20.}}	{6.77873, 31.0003}	inf	238.	510.799	{407.604, 627.442}	114.621	0.32513	newly	95.	168.13	{141., 198.}	76.9789	0.243993	Reff	0.382254	0.400513	{0.369297, 0.41716}	6.11176	ar	8478.	9481.79	{9274., 9672.}	11.8399
5	R0	{2.96649, 18.0674}	{2.79369, 19.7156}	{{2.46114, 17.7713}, {2.89342, 20.}}	{5.82493, 9.9177}	inf	369.	507.25	{451.716, 551.801}	37.466	0.136507	newly	132.	155.38	{139., 172.}	17.9848	0.070597	Reff	0.177989	0.223963	{0.204885, 0.285632}	25.8297	ar	9476.	10 761.8	{10 502., 10 929.}	13.5687
6	R0	{2.94778, 16.0002}	{2.77358, 19.6899}	{{2.51324, 18.4266}, {2.93603, 20.}}	{6.15433, 23.2141}	inf	276.	619.857	{541.113, 679.507}	124.586	0.348671	newly	93.	191.43	{167., 213.}	105.839	0.311514	Reff	0.21961	0.24574	{0.224822, 0.285467}	11.9248	ar	9321.	10 644.6	{10 376., 10 791.}	14.2
7	R0	{2.17562, 11.7184}	{2.62476, 17.7135}	{{2.24405, 13.9255}, {3., 20.}}	{21.0748, 51.1595}	inf	199.	555.026	{338.131, 699.331}	178.908	0.428318	newly	97.	187.11	{141., 222.}	92.8969	0.278878	Reff	0.392917	0.32979	{0.258031, 0.413114}	19.2022	ar	8345.	10 416.1	{9882., 10 846.}	24.8183
8	R0	{2.7904, 17.8966}	{2.33334, 19.4121}	{{2.11914, 16.9923}, {2.44543, 20.}}	{16.3799, 10.14}	inf	456.	635.14	{525.784, 684.768}	39.2851	0.142005	newly	172.	202.04	{180., 225.}	17.6395	0.0693053	Reff	0.233277	0.343808	{0.309949, 0.397811}	47.3816	ar	9285.	10 072.8	{9630., 10 299.}	8.48422
9	R0	{2.51574, 16.376}	{2.52287, 19.8083}	{{2.34726, 20.}, {2.76943, 20.}}	{4.34644, 20.9592}	inf	368.	608.696	{545.1, 665.366}	65.4064	0.217318	newly	131.	188.76	{159., 215.}	44.0916	0.156127	Reff	0.296857	0.314595	{0.257951, 0.355293}	10.4117	ar	8937.	10 508.8	{10 272., 10 699.}	17.5872
10	R0	{2.56405, 18.1787}	{2.21893, 18.3102}	{{2.12402, 17.5123}, {2.43348, 20.}}	{13.4644, 3.80652}	inf	542.	650.764	{595.15, 715.747}	20.0671	0.0781149	newly	185.	222.02	{202., 248.}	20.2919	0.0790439	Reff	0.307686	0.395784	{0.330541, 0.422197}	28.8225	ar	9005.	10 190.	{9964., 10 575.}	13.1591
11	R0	{2.475, 17.1709}	{1.94839, 20.}	{{1.92692, 20.}, {1.97394, 20.}}	{21.2771, 16.4761}	inf	441.	714.611	{694.269, 737.978}	62.0434	0.209483	newly	155.	228.37	{211., 245.}	47.3355	0.167499	Reff	0.328185	0.496937	{0.485892, 0.507301}	51.4197	ar	8870.	9444.88	{9362., 9536.}	6.48117
12	R0	{2.14974, 11.8619}	{2.25063, 18.6953}	{{2.11055, 17.4758}, {2.45781, 20.}}	{5.32278, 57.6083}	inf	247.	685.101	{624.781, 725.33}	177.369	0.441529	newly	113.	227.11	{204., 256.}	100.982	0.301477	Reff	0.367391	0.383041	{0.322651, 0.425808}	10.9219	ar	8414.	10 027.8	{9774., 10 331.}	19.1802
13	R0	{2.20385, 14.4767}	{2.19567, 20.}	{{2.02681, 20.}, {2.25829, 20.}}	{2.50359, 38.153}	inf	257.	546.095	{505.39, 608.037}	112.488	0.326102	newly	114.	172.32	{154., 198.}	51.1579	0.17738	Reff	0.397134	0.42756	{0.408701, 0.465213}	7.66139	ar	8347.	9824.13	{9574., 9982.}	17.6965
14	R0	{2.35671, 13.8357}	{2.5731, 18.2281}	{{2.17842, 14.9705}, {2.91301, 20.}}	{13.9269, 32.4269}	inf	237.	478.037	{327.746, 598.493}	102.283	0.292466	newly	104.	158.2	{131., 184.}	52.1346	0.177693	Reff	0.328997	0.340501	{0.263711, 0.435195}	19.6278	ar	8719.	10 199.7	{9624., 10 623.}	16.9825
15	R0	{1.91368, 10.1082}	{2.13489, 18.6533}	{{2.09174, 18.104}, {2.22039, 20.}}	{11.5879, 84.5364}	inf	135.	547.936	{503.514, 665.04}	305.879	0.605944	newly	76.	183.56	{165., 208.}	141.526	0.381088	Reff	0.503681	0.423892	{0.408136, 0.430576}	15.9814	ar	7486.	9747.17	{9635., 9973.}	30.2053
16	R0	{2.48727, 14.882}	{2.81574, 19.9438}	{{2.5594, 20.}, {2.89675, 20.}}	{13.5599, 34.0131}	inf	327.	615.316	{544.221, 668.674}	88.1701	0.273372	newly	131.	186.53	{164., 206.}	42.3893	0.15153	Reff	0.299467	0.216568	{0.194746, 0.268867}	28.3753	ar	8894.	11 044.	{10 916., 11 164.}	24.1734
17	R0	{2.46795, 13.1915}	{2.87129, 18.9855}	{{2.33708, 15.5771}, {3., 20.}}	{18.0651, 44.1715}	inf	235.	522.058	{359.774, 602.856}	122.152	0.338771	newly	107.	163.66	{132., 187.}	52.9533	0.181077	Reff	0.326757	0.220699	{0.180425, 0.306532}	33.1186	ar	8796.	10 559.8	{10 176., 10 731.}	20.0521
18	R0	{2.96603, 16.7543}	{2.8311, 19.9981}	{{2.77027, 20.}, {2.90289, 20.}}	{4.5492, 19.3613}	inf	304.	531.952	{500.189, 565.894}	74.9842	0.242403	newly	107.	162.05	{142., 181.}	51.4486	0.178508	Reff	0.209995	0.21332	{0.203207, 0.222597}	3.25126	ar	9394.	10 718.2	{10 615., 10 852.}	14.0966
19	R0	{2.25216, 9.90465}	{2.93929, 19.5463}	{{2.5551, 16.6349}, {3., 20.}}	{30.5097, 97.3446}	inf	114.	606.172	{522.918, 665.958}	431.73	0.723855	newly	62.	186.63	{163., 207.}	201.016	0.476779	Reff	0.321383	0.209108	{0.184253, 0.28243}	34.935	ar	8631.	11 178.1	{11 068., 11 280.}	29.5114
20	R0	{2.70837, 18.1923}	{2.47488, 19.8089}	{{2.29512, 20.}, {2.56865, 20.}}	{8.62104, 9.42943}	inf	470.	674.624	{623.661, 718.186}	43.5371	0.156101	newly	154.	212.04	{188., 238.}	37.6883	0.137071	Reff	0.256212	0.312781	{0.286419, 0.359715}	22.0791	ar	9183.	10 519.3	{10 335., 10 658.}	14.5517
21	R0	{2.30288, 15.5877}	{2.20729, 20.}	{{2.16948, 20.}, {2.2426, 20.}}	{4.15083, 28.3063}	inf	338.	648.844	{590.018, 686.979}	91.9658	0.282292	newly	137.	203.84	{180., 225.}	48.7883	0.170602	Reff	0.344741	0.435529	{0.422733, 0.448976}	26.3351	ar	8674.	9777.62	{9665., 9890.}	12.7233
22	R0	{2.82208, 10.7544}	{2.63641, 15.5642}	{{2.60759, 15.2864}, {2.66252, 15.7816}}	{6.57908, 44.7241}	inf	91.	342.023	{323.909, 360.308}	275.849	0.574404	newly	39.	130.6	{116., 144.}	234.872	0.52325	Reff	0.196981	0.246847	{0.243483, 0.250392}	25.3151	ar	9333.	10 258.9	{10 188., 10 314.}	9.9206
23	R0	{1.69923, 10.1366}	{2.25792, 19.0186}	{{1.85204, 16.3483}, {2.42841, 20.}}	{33.2444, 87.623}	inf	136.	636.013	{523.186, 719.36}	367.657	0.663259	newly	84.	209.3	{170., 235.}	149.167	0.393548	Reff	0.525912	0.471264	{0.42067, 0.587062}	15.6155	ar	7055.	10 196.3	{9441., 10 572.}	44.5263
24	R0	{2.64944, 19.054}	{2.16944, 19.9039}	{{2.13885, 20.}, {2.19578, 20.}}	{18.1172, 5.07191}	inf	411.	535.464	{505.462, 561.653}	30.9104	0.116511	newly	150.	167.81	{147., 184.}	13.6067	0.0548659	Reff	0.26097	0.384118	{0.374622, 0.394416}	47.1885	ar	9147.	9843.74	{9730., 9929.}	7.61714
25	R0	{2.53191, 10.9214}	{2.48194, 19.9986}	{{2.44727, 20.}, {2.50489, 20.}}	{1.97347, 83.1141}	inf	104.	617.566	{601.205, 629.707}	493.813	0.773528	newly	53.	189.69	{176., 204.}	257.906	0.552932	Reff	0.259774	0.304031	{0.297986, 0.31523}	17.0368	ar	9009.	10 117.7	{10 044., 10 190.}	12.3064
26	R0	{1.89155, 6.76944}	{2.9475, 18.162}	{{2.71121, 16.6611}, {3., 18.7049}}	{55.8244, 168.295}	inf	18.	407.055	{379.827, 443.911}	2161.42	1.35311	newly	10.	135.42	{121., 152.}	1254.2	1.13012	Reff	0.5058	0.250115	{0.234415, 0.2901}	50.5507	ar	7348.	10 700.8	{10 519., 10 818.}	45.6287
27	R0	{2.63149, 15.6979}	{2.70718, 19.9527}	{{2.53384, 20.}, {2.85411, 20.}}	{5.43046, 27.1587}	inf	374.	645.06	{583.489, 705.613}	72.476	0.235316	newly	136.	197.26	{173., 218.}	45.0441	0.159754	Reff	0.26236	0.245243	{0.213198, 0.281612}	11.6855	ar	9104.	10 875.6	{10 769., 10 990.}	19.46
28	R0	{2.85141, 12.6992}	{2.79186, 16.5807}	{{2.69348, 16.0352}, {3., 18.1154}}	{3.33895, 30.5651}	inf	136.	359.954	{326.705, 392.782}	164.672	0.420361	newly	59.	128.49	{112., 142.}	117.78	0.335992	Reff	0.201595	0.21004	{0.17863, 0.222745}	7.12203	ar	9317.	10 657.4	{10 558., 10 775.}	14.3866
29	R0	{2.54244, 12.9085}	{2.10451, 17.7111}	{{2.05012, 17.3459}, {2.12798, 17.7994}}	{17.2247, 37.2046}	inf	177.	517.022	{487.781, 566.09}	192.103	0.464113	newly	83.	180.63	{163., 198.}	117.627	0.336437	Reff	0.256278	0.408381	{0.397742, 0.427545}	59.3508	ar	9034.	9563.11	{9412., 9650.}	5.85687
30	R0	{2.10982, 11.7636}	{2.15218, 18.5432}	{{2.06078, 17.4115}, {2.26192, 20.}}	{3.56597, 57.6316}	inf	182.	587.897	{513.173, 659.99}	223.02	0.50667	newly	78.	196.87	{173., 216.}	152.397	0.400404	Reff	0.400444	0.404098	{0.370094, 0.424495}	5.11501	ar	8224.	9872.88	{9613., 10 177.}	20.0496
31	R0	{2.76312, 15.5396}	{2.66237, 18.2573}	{{2.30834, 14.8059}, {3., 20.}}	{8.78308, 20.9095}	inf	308.	458.085	{296.088, 600.459}	52.9508	0.177912	newly	111.	150.03	{116., 180.}	36.4955	0.130811	Reff	0.234589	0.252563	{0.183181, 0.317802}	19.1635	ar	9220.	10 374.5	{9923., 10 724.}	12.5216
32	R0	{2.21727, 15.5439}	{1.84045, 20.}	{{1.78715, 20.}, {1.93429, 20.}}	{16.9948, 28.6678}	inf	307.	632.282	{527.84, 684.442}	105.955	0.311514	newly	111.	203.35	{167., 230.}	83.1982	0.259721	Reff	0.413964	0.541957	{0.489021, 0.569097}	30.9187	ar	8359.	9369.91	{9137., 9721.}	12.0937
33	R0	{2.72912, 10.8159}	{3., 20.}	{{3., 20.}, {3., 20.}}	{9.92554, 84.913}	inf	49.	437.303	{429.497, 461.024}	792.454	0.950464	newly	25.	131.57	{117., 144.}	426.28	0.719848	Reff	0.222969	0.164717	{0.162162, 0.167509}	26.1257	ar	9196.	10 833.9	{10 813., 10 869.}	17.8107
34	R0	{2.20928, 10.1308}	{2.55986, 14.6947}	{{1.95663, 9.42952}, {3., 19.6011}}	{18.4978, 47.1043}	inf	59.	269.753	{117.845, 468.886}	357.209	0.630517	newly	37.	105.44	{82., 144.}	184.973	0.445339	Reff	0.360775	0.327872	{0.21807, 0.340505}	19.2495	ar	8388.	10 346.2	{9055., 10 922.}	23.345
35	R0	{2.10103, 15.2673}	{2.16053, 18.5626}	{{2.05583, 17.2576}, {2.30884, 20.}}	{4.05427, 21.5843}	inf	355.	554.819	{466.766, 697.1}	56.2872	0.189854	newly	143.	184.84	{159., 213.}	29.2587	0.108875	Reff	0.409701	0.397499	{0.354426, 0.426258}	5.37205	ar	8264.	9705.7	{9435., 10 027.}	17.4455
36	R0	{2.74914, 19.2775}	{2.2612, 20.}	{{2.21995, 20.}, {2.30139, 20.}}	{17.7489, 3.74789}	inf	508.	662.078	{626.804, 691.646}	30.3303	0.11441	newly	161.	206.75	{184., 229.}	28.4161	0.106985	Reff	0.249622	0.41728	{0.405136, 0.429706}	67.1649	ar	9301.	10 210.2	{10 099., 10 313.}	9.77572
37	R0	{2.78161, 16.0136}	{2.74833, 19.992}	{{2.51976, 20.}, {2.85516, 20.}}	{4.06877, 24.8438}	inf	295.	559.408	{510.083, 655.204}	89.6297	0.275246	newly	117.	172.18	{145., 208.}	47.1624	0.164197	Reff	0.232264	0.262861	{0.23834, 0.31357}	13.1731	ar	9240.	10 912.7	{10 766., 11 035.}	18.1028
38	R0	{2.4454, 8.33501}	{3., 20.}	{{3., 20.}, {3., 20.}}	{22.6793, 139.952}	inf	27.	570.238	{556.043, 587.681}	2011.99	1.3246	newly	18.	171.61	{153., 187.}	853.389	0.978066	Reff	0.282933	0.164766	{0.1609, 0.168536}	41.7651	ar	8853.	11 084.6	{11 055., 11 113.}	25.2077
39	R0	{2.9154, 12.3861}	{2.86073, 18.1825}	{{2.69863, 15.8778}, {3., 20.}}	{4.77712, 46.7979}	inf	88.	426.106	{289.526, 543.655}	384.211	0.669348	newly	42.	138.25	{102., 171.}	229.167	0.508398	Reff	0.190376	0.224694	{0.19032, 0.265802}	18.2135	ar	9371.	10 482.7	{10 267., 10 674.}	11.8633
40	R0	{2.40923, 15.1963}	{2.44314, 19.7351}	{{2.21553, 18.0647}, {2.57705, 20.}}	{4.0558, 29.8679}	inf	294.	634.379	{578.276, 688.006}	115.775	0.33286	newly	115.	199.64	{181., 224.}	73.6	0.237898	Reff	0.339701	0.324384	{0.284174, 0.381764}	9.61525	ar	8735.	10 585.6	{10 195., 10 761.}	21.1858
41	R0	{2.63137, 15.238}	{2.25536, 18.5643}	{{2.14274, 17.3845}, {2.47714, 20.}}	{14.2896, 21.8292}	inf	281.	520.113	{464.189, 597.407}	85.0936	0.265489	newly	89.	172.45	{151., 195.}	93.764	0.285194	Reff	0.24998	0.367975	{0.301427, 0.398126}	47.2019	ar	9150.	9951.21	{9693., 10 292.}	8.75639
42	R0	{2.48769, 19.4593}	{1.97194, 20.}	{{1.93405, 20.}, {2.01017, 20.}}	{20.7319, 2.77862}	inf	467.	531.803	{503.316, 558.042}	14.024	0.0560447	newly	165.	169.91	{149., 193.}	8.50303	0.0355622	Reff	0.312454	0.472728	{0.452346, 0.493558}	51.2952	ar	8932.	9763.42	{9561., 9930.}	9.30833
43	R0	{2.61199, 10.214}	{2.81724, 17.803}	{{2.6758, 16.2879}, {3., 20.}}	{7.85818, 74.2999}	inf	68.	535.104	{433.05, 702.483}	686.918	0.886675	newly	40.	179.45	{152., 214.}	348.625	0.647974	Reff	0.251796	0.267376	{0.207745, 0.30544}	17.4777	ar	9061.	10 506.9	{10 301., 10 808.}	15.9573
44	R0	{2.50994, 18.3382}	{1.93107, 20.}	{{1.91001, 20.}, {1.94807, 20.}}	{23.0633, 9.06196}	inf	483.	693.797	{670.032, 725.68}	43.6432	0.156876	newly	173.	221.15	{204., 238.}	27.8324	0.105668	Reff	0.350137	0.499834	{0.489152, 0.511691}	42.754	ar	8827.	9325.34	{9207., 9438.}	5.64563
45	R0	{2.44283, 16.8338}	{2.24423, 19.0814}	{{2.10696, 17.6481}, {2.38437, 20.}}	{8.12983, 13.3514}	inf	399.	580.2	{511.848, 649.726}	45.4135	0.160665	newly	142.	187.25	{166., 207.}	31.8662	0.118489	Reff	0.308285	0.361333	{0.319375, 0.398639}	17.2125	ar	8878.	10 181.7	{9885., 10 478.}	14.6848
46	R0	{2.51815, 18.1462}	{2.22628, 20.}	{{2.09904, 20.}, {2.27716, 20.}}	{11.5908, 10.2159}	inf	537.	638.706	{562.01, 687.81}	19.1106	0.0746019	newly	173.	199.21	{170., 225.}	17.0578	0.0674771	Reff	0.31905	0.418128	{0.399863, 0.440674}	31.0541	ar	8978.	9973.44	{9809., 10 111.}	11.0875
47	R0	{2.88885, 16.8987}	{2.1022, 18.4106}	{{2.03127, 17.4684}, {2.20657, 20.}}	{27.2307, 8.94694}	inf	414.	632.953	{563.231, 792.395}	52.8871	0.180695	newly	129.	214.25	{190., 242.}	66.0853	0.218391	Reff	0.22533	0.423865	{0.393064, 0.439055}	88.1084	ar	9337.	9584.32	{9375., 9832.}	2.70601
48	R0	{2.58709, 13.8786}	{2.86075, 19.8239}	{{2.8434, 20.}, {2.92107, 20.}}	{11.3617, 42.8382}	inf	256.	602.227	{560.753, 651.813}	135.264	0.37006	newly	109.	183.84	{163., 201.}	68.6606	0.225659	Reff	0.243704	0.212514	{0.200432, 0.21362}	15.6848	ar	9146.	11 003.9	{10 898., 11 104.}	20.3136
49	R0	{2.42959, 10.7132}	{2.97889, 19.8323}	{{3., 20.}, {3., 20.}}	{22.9377, 85.1206}	inf	116.	566.301	{560.448, 593.66}	388.19	0.687221	newly	60.	172.78	{156., 189.}	187.967	0.457941	Reff	0.288149	0.177141	{0.169455, 0.178275}	38.9611	ar	8863.	10 949.3	{10 850., 11 094.}	23.5394
50	R0	{2.60513, 13.2796}	{2.765, 20.}	{{2.72413, 20.}, {2.80087, 20.}}	{6.1368, 50.6069}	inf	243.	653.559	{644.925, 663.678}	168.954	0.429646	newly	108.	198.12	{184., 212.}	83.4444	0.262927	Reff	0.256605	0.24584	{0.239047, 0.252879}	4.23113	ar	9095.	10 639.1	{10 507., 10 741.}	16.9772
